# Circulating Endothelial Microparticles: A Key Hallmark of Atherosclerosis Progression

**DOI:** 10.1155/2016/8514056

**Published:** 2016-03-15

**Authors:** Keshav Raj Paudel, Nisha Panth, Dong-Wook Kim

**Affiliations:** ^1^Department of Oriental Medicine Resources, Mokpo National University, Muan-gun, Jeonnam 534-729, Republic of Korea; ^2^College of Pharmacy and Natural Medicine Research Institute, Mokpo National University, Muan-gun, Jeonnam 58554, Republic of Korea

## Abstract

The levels of circulating microparticles (MPs) are raised in various cardiovascular diseases. Their increased level in plasma is regarded as a biomarker of alteration in vascular function. The prominent MPs present in blood are endothelial microparticles (EMPs) described as complex submicron (0.1 to 1.0 *μ*m) vesicles like structure, released in response to endothelium cell activation or apoptosis. EMPs possess both physiological and pathological effects and may promote oxidative stress and vascular inflammation. EMPs release is triggered by inducer like angiotensin II, lipopolysaccharide, and hydrogen peroxide leading to the progression of atherosclerosis. However, there are multiple physiological pathways for EMPs generation like NADPH oxidase derived endothelial ROS formation, Rho kinase pathway, and mitogen-activated protein kinases. Endothelial dysfunction is a key initiating event in atherosclerotic plaque formation. Atheroemboli, resulting from ruptured carotid plaques, is a major cause of stroke. Increasing evidence suggests that EMPs play an important role in the pathogenesis of cardiovascular disease, acting as a marker of damage, either exacerbating disease progression or triggering a repair response. In this regard, it has been suggested that EMPs have the potential to act as biomarkers of disease status. This review aims to provide updated information of EMPs in relation to atherosclerosis pathogenesis.

## 1. What Are Microparticles?

Microparticles (MPs) are cell membrane-shedded submicron fragments ranging from 0.1 to 1.0 *μ*m containing information like mRNA, microRNAs (miRNAs), receptor, and specific proteins of parent cell. MPs are expressed by cells during cellular stress and cell activation [1]. To date, MPs have been identified as membrane vesicles released from blood-related cells such as endothelial cells, smooth muscle cells, platelets, erythrocytes, and leukocytes, as recognized by their specific surface proteins [2, 3]. In history, MPs were regarded as “cell dust/debris” and the process of their formation remains to be completely elucidated [4, 5]. However, it is clear that MPs formation and shedding involve reconstitution of cell membrane phospholipid structure, with outer leaflet subjected to phosphatidylserine (PS), and alteration of the cell architecture, with the disruption of cytoskeleton organization [6–9]. Several studies report the existence of small vesicles similar in size as MPs but do not expose PS in the outer leaflet. The area of MP study is rapidly growing as the role of MPs in the pathogenesis of diabetes mellitus [10], lungs cancer [11, 12], central nervous system disorder [13], inflammation [14, 15], systemic lupus erythematosus [16], and cardiovascular diseases (CVDs) [17] has been highlighted by various clinical and experimental research.

## 2. Types of Microparticles

The types of microparticles and their role in disease progression are summarized in Table 1.

## 3. Isolation of Microparticles

Although, some of the research studies on MPs derived* ex vivo* from blood of suitable patient, but due to the less number of MPs derived from this methods, various* in vitro* cells lines (e.g., Jurkat cells and human umbilical vein endothelial cells) stimulated with cytokine, chemicals, or apoptosis inducer are use in research laboratory to obtain MPs. Various inducers like angiotensin II, lipopolysaccharide, and hydrogen peroxide can be applied to generate MPs* in vitro*.

In brief, the MPs containing the suspension of cell, treated with suitable stimulus, are subjected to initial low speed centrifugation at ~1500 ×g to remove the cells followed by second high speed centrifugation at ~20,000 ×g to get MPs pellet at the bottom. After removing the supernatant, these MPs are suspended on saline buffer solution, divided as necessary, and stored at −70–80°C until further use. In contrast to this method, some researchers also used MPs pellets after ultracentrifugation at a speed of 100,000 ×g. In this case, not only MPs, but also exosomes vesicles of diameter 40–90 nm settle down at the bottom as sediment [18].

## 4. What Are Endothelial Microparticles?

In human blood circulation, endothelial MPs (EMPs) constitute a smaller population of MP family. However, it has been widely associated with pathogenesis of various CVDs, mainly initiated by endothelial dysfunction [22, 45, 46]. Researches have demonstrated the key role of EMPs in the growth, division, and maturation of endothelial precursor cells, important for blood vessel regeneration [47], suggesting a potential preservative role in response to vascular regeneration, restoration, and protection [48]. It is of great interest to know the difference in the level of EMPs in healthy and pathological condition. In this aspect, Mezentsev et al. [49] have carried out experiment to highlight the various designs of angiogenesis (cell proliferation rate, capillary formation, and death of endothelial cells) by* in vitro* assay, quantifying physiological levels of EMPs in healthy volunteers (ranging from 10^3^ to 10^4^ EMPs/mL) [33, 50] and diseased state concentrations (measured in person with cardiovascular disease, 10^5^ EMPs/mL) [51–53]. Higher levels of EMPs are believed to affect angiogenesis, with the amplitude of the effect being directly proportional to EMPs concentration. Another study by Koga et al. found the presence of cluster of differentiation (CD) 144 also known as vascular endothelial- (VE-) cadherin positive EMPs in human circulation, with higher levels being seen in those suffering from diabetic mellitus and coronary artery disease including atherosclerosis. Consequently, VE-cadherin positive EMPs can be a hallmark for analyzing atherosclerosis* via* endothelial cell dysfunction. Taken together, various cardiovascular complications can be efficiently prevented by therapeutics approach that target atherosclerosis patients focusing on assessments of blood CD144-EMP levels [34].

## 5. What Is Atherosclerosis?

**Figure 1:**
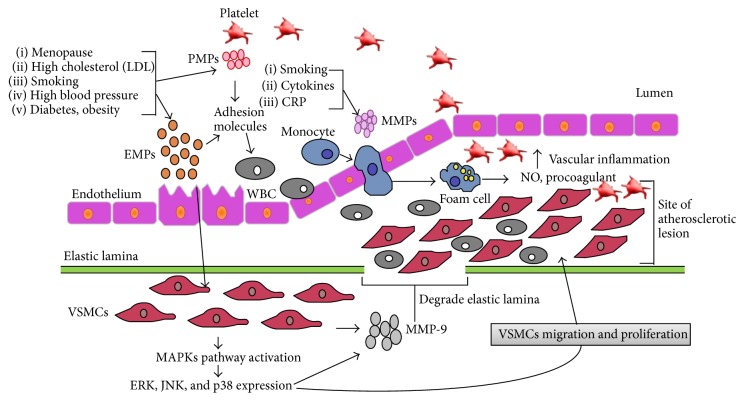
The chain of events initiated by MPs on atherosclerosis physiopathology. Endothelial microparticles (EMPs) and platelet microparticles (PMPs) are generated by the activation of endothelium with a number of factors like smoking, oxidative stress, obesity, and high blood pressure whereas monocyte-microparticles (MMPs) are generated by proinflammatory cytokines (tumor necrosis factor-*α*, interleukin 1-*β*), cigarette smoking, and C-reactive protein (CRP). EMPs can activate vascular smooth muscle cells (VSMCs) to express matrix metalloproteinase-9 (MMP-9) that can degrade elastic lamina barrier to facilitate VSMC migration from tunic media to tunic intima at the site of atherosclerotic lesion. Furthermore, EMPs also upregulate ERK, JNK, and p38 through MAPKs pathway leading to proliferation and migration of VSMC to develop atheroma plaque. Cell adhesion molecules expressed by EMPs and PMPs facilitate leukocytes and platelets adhesion to the endothelial surface thereby developing intimal thickening and vascular lesion. MMPs can activate inducible nitric oxide synthase pathway to release nitric oxide (NO) and procoagulant responsible for vascular inflammation and endothelial dysfunction. Collectively, all this event leads to atherosclerosis.

Atherosclerosis is described as a vascular inflammatory disorder characterized by narrowing of blood vessel lumen due to accumulation of lipid, inflammatory cell, vascular smooth muscle cell (VSMC), and platelet triggered by lipid peroxidation, endothelial dysfunction, release of inflammatory mediators by activated macrophage, and VSMC migration and proliferation in intimal layer [54, 55]. It is initiated by endothelial dysfunction whereby endothelial cells produce chemoattractant factors that induce monocyte recruitment and infiltration in the neointima. At intimal site, monocytes differentiate into macrophages and engulf oxidized low-density lipoproteins (LDLs) derived from the circulation to the site of lesion, leading to the formation of inflammatory foam cells. Atherosclerotic lesion progresses with exacerbated macrophage accumulation and release of various inflammatory mediators such as cyclooxygenase-2 (COX-2), inducible nitric oxide synthase (iNOS), tumor necrosis factor (TNF-*α*), and interleukin followed by VSMC migration and proliferation from the media to the intima due to the activation of mitogen-activated protein kinases (MAPKs) pathways, collagen production, and lesion calcification [56, 57]. Atheroma buildup is facilitated by platelet and other circulating cell accumulation. Eventually, the brust of the atherosclerotic plaque due to high blood pressure results in the occlusion of artery lumen mainly in small artery with narrow lumen and further complication. Atherosclerosis develops quietly many years ago before clinical manifestation is revealed. However, early identification of atheroma formation may allow measures to prevent the progression of the acute condition towards serious clinical events (Figure 1). Since imaging techniques do not allow early detection and cannot be used routinely, finding circulating biomarkers is of great importance to predict cardiovascular disease risk and pathology prognosis [58, 59].

## 6. Role of EMPs on Pathogenesis of Atherosclerosis

The scientific highlight about the role of MPs on atherosclerosis progression is summarized in Table 2.

### 6.1. Association of Oxidative Stress with EMPs

The role of oxidative stress as a stimulus for EMPs release facilitating atherosclerosis progression was described by Szotowski et al. and Vince et al. According to the study done by Szotowski et al., the evidence of the relationship among reactive oxygen spices (ROS) formation and production of EMP-associated tissue factor was established. Furthermore, the pretreatment of any antioxidant compound before application of ionization radiation (source of ROS formation) and TNF-*α* induction to endothelial cell remarkably inhibited ROS formation. This inhibition of ROS production was accompanied with a lower expression of thrombogenic EMPs, highlighting the positive relationship of ROS and the formation of thrombogenic EMPs [35]. When cells are lacking enough oxygen, they are metabolically impaired resulting in compromised cellular function eventually leading to apoptosis* via* metabolic failure. Apoptosis is a potent signal for EMPs generation [1]. Polymorphoneutrophils (PMNs) bound to the endothelial surface upregulate endothelial dysfunction and histological damage through a variety of noxious pathways. At this point, activated neutrophils moderate a variety of deleterious responses like generation of ROS, cytotoxic enzymes, and inflammation mediators or cytokines. Moreover, they also hire additional neutrophils by chemotaxis and enhance their attachment to the vascular intimal endothelium though adhesion glycoprotein molecules were expressed on both the surfaces of the neutrophil and endothelial cell. High levels of ROS can distort the redox equilibrium of cells causing oxidative stress and destruction to cell membrane organization followed by membrane microparticle release, protein degradation, and apoptosis [15].

### 6.2. Monocyte-Macrophage MPs in Atherosclerosis

In patients with atherosclerosis, inflammatory macrophage pools are enlarged in the atherosclerotic plaques. Monocyte-macrophage-derived MPs are critical part of the progression of unstable atherosclerotic plaques. Monocyte-macrophages express MPs on activation by cigarette consumption, proinflammatory cytokines (TNF-*α*, IL-1*β*) and C-reactive protein. Apart from this, endotoxin induces macrophage MPs formation via a pathway essential for inducible nitric oxide synthase (iNOS) activation [40]. It is well established that iNOS overexpression greatly influence vascular inflammation by the generation of significant amount of nitic oxide (NO) [54]. Furthermore, tobacco smoke provokes the generation of highly procoagulant monocytic MPs in a process requiring extracellular signal-regulated kinases (ERK1/ERK2) activation [41]. ERK activation is a factor causing VSMC proliferation in atherosclerosis [55]. The MPs originated from monocytes contain procoagulant substances responsible for endothelial dysfunction [25]. A research done by Wang et al. showed that the presence of IL-1*β* in monocyte-macrophage MPs can enhance the vascular inflammatory process and activate endothelial cells [42]. Similarly, Hoyer et al. demonstrated the role of monocyte-macrophage MPs in atherosclerotic vascular inflammation and showed that the application of monocyte-macrophage MPs in ApoE^−/−^ mice facilitated the development of atherosclerotic plaque in the gene knock-out mice and also increase the buildup of macrophages in the vessel wall. This study has clarified crucial interaction between monocyte-macrophage EMPs and vascular inflammatory in the atherosclerotic disease progression of ApoE^−/−^ mice [27]. Human atherosclerotic plaques contain MPs released from various apoptotic cell deaths within the necrotic lesion site. Milbank et al. investigated the role of MPs generated by apoptotic murine macrophage (RAW 264.7) cell in adult murine cardiomyocytes. The results showed that MPs contained the soluble form of TNF-*α*. After application of MPs, there was increased expression of apoptosis regulator bcl-2-like protein 4 (Bax), caspase-8, caspase-3, and cytochrome C in cardiomyocytes. These results provide evidence that MPs released from activated macrophages possessing TNF-*α* could contribute to the promotion of vascular inflammatory signals leading to atherosclerosis and myocardial infraction [43].

### 6.3. EMPs and Matrix Metalloproteinase Association in Atherosclerosis

Considerable research favours that microparticles could induce expression of metalloproteinases (MMPs), thereby degrading extracellular matrix (ECM) component by its proteolytic activity [60, 61]. EMPs play an important role in plasmin (enzyme needed to activate MMPs) localization and activation. MMP-2 and MMP-9 functions are affected by MPs produced by cell other than endothelial cells [60, 62]. For atherosclerosis event, MMP-9 and MMP-2 play key role by degrading ECM and facilitating VSMC migration from media to intima of blood vessel [54]. Lozito and Tuan disclose the endogenous MMPs' proteolytic activity of EMPs by expressing both membrane-type MMPs and soluble molecules believed to be crucial in maintaining ECM. EMPs can activate endogenous zymogen, proMMP-2, a feature not seen in the remaining endothelial cell secreted factors to provide the evidence of MP-induction of MMP-2 with MMP-14. Endothelial cell secreted factors include the increasing concentration of tissue inhibitors of metalloproteinase-1 (TIMP-1) and TIMP-2, which were not detected in MPs [36]. The release of ECM cleaving proteases, particularly MMPs by endothelial cells, is an important process during angiogenesis and atherosclerosis. Taraboletti et al. found that* in vitro* cultured human umbilical vein endothelial cells (HUVEC) shed microparticles in the range of 0.3–0.6 *μ*m from the cells plasma membrane, as observed by ultrastructural analysis. Detailed analysis of microparticles showed that the two principle gelatinases essential for atherosclerosis progression, MMP-2 and MMP-9, were discovered on the outer side of the microparticles membrane. It is clear that endothelial cells upon stimulation by various means shed MMP-containing microparticles and this is responsible for regulating proteolytic activity of MMPs to degrade the ECM and promote VSMC migration for atheroma formation in neointima [60].

### 6.4. EMPs Linked with MAPKs Signaling Pathway

MAPK is a cell signaling pathway directly related to atherosclerosis pathophysiology as it induces proliferation and migration of vascular smooth muscle cell [53, 55]. MAPK is a family of three proteins, namely, extracellular signal-regulated kinases (ERK), c-*jun* terminal NH_2_-kinase (JNK), and p38 [56]. Previous researches have demonstrated the activation of different cell signaling pathways during EMPs release.* In vitro* experiment has shown that proinflammatory mediators can cause endothelial apoptosis and activation, thereby increasing EMPs basal release through MAPKs dependent pathways [48]. p38-MAPK pathways are involved in controlling the TNF-*α* dependent EMPs release [37, 63]. It was observed that reducing the expression of p38-MAPK in human aortic endothelial cells, using therapeutics agents, caused the reduction of TNF-*α*-induced EMPs by the half. It is interesting to note that the suppression of MPs was focused on p38 MAPK pathways. These findings revealed p38 MAPK signaling as potent and specific in the generation and maturation of EMPs and as an unidirectional model where p38 MAPK serves as critical source of microparticle formation, unlike the target cellular response to EMPs [37]. Likewise, it also proposes a new pathway which offer* in vivo* therapeutic benefit* via* direct inhibition of EMP formation by p38 inhibition. [37]. In physiological system, during the initial events of atherosclerosis progression, intense tissue factor (TF) generation is seen in monocytes. However, at later advance stages of atherosclerosis, TF release is also observed in modified macrophage (foam cells), endothelial cells, and VSMC [64]. TF exists in the necrotic lesion of atheroma plaques as well, particularly linked with MPs derived from apoptotic foam cells, macrophages, or lymphocytes. These MPs of course exhibit the TF activity in atherosclerotic plaques [38]. TF bearing microparticles are released by endothelial cell induced by cytokines such as TNF-*α*, IL-1*β*, [65] and the MAPKs p38, ERK, and JNK are involved in TNF-*α*-induced TF expression [66].

### 6.5. EMPs Induced Vascular Inflammation

The influence of MPs on vascular inflammation to cause atherosclerosis has been studied in detail. Considerable studies favour a role for MPs in upregulating proinflammatory mediators like IL-6, monocyte chemoattractant protein-1 (MCP-1), inducible nitric oxide synthase (iNOS), and cyclooxygenase-2 (COX-2) release by endothelial cells following JNK1 and nuclear factor kappaB (NF-*κ*B) pathways [67, 68] and TNF-*α* and IL-1 by monocytes [69]. Arachidonic acid, a marker of cell injury/inflammation, secreted from platelet-derived MPs, enhances COX-2 and intracellular adhesion molecules-1 (ICAM-1) release in endothelial cells as well as COX-2 expression in monocytes through the translocation of protein kinase C (PKC) from the cytosol to the cell membrane [28, 29]. Furthermore, aminophospholipids placed on the MPs membrane can also boost inflammation. Aminophospholipids act as substrates for phospholipase A2, responsible for catalyzing the production of lysophosphatidic acid (LPA), a factor of vascular inflammation [70]. EMPs release is also induced by thrombin mediated inflammatory particles like IL-8 and interleukin-1 receptor antagonists (IL-1Ra) and IL-1 that are involved in blood coagulation, vascular inflammation, and angiogenesis [71, 72]. Vascular inflammation and coagulation are linked processes as they collectively build up atheroma in blood vessel wall, and, in this step, MPs may intensify the responses by activating NF-*κ*B, elevating nitric oxide (NO) release, and increasing oxidative stress [9, 73]. Scientific evidence evinces that MPs induce harmful effects on vascular function* via* the release of inflammatory cytokines/chemokines as well as increased expression of endothelial cell adhesion molecules [29, 30, 74]. MPs obtained from white blood cells (WBC) also circulate along with blood at low levels in individuals with good health and are at high levels during vascular inflammation [67].* In vitro* experiment revealed that such MPs can activate endothelial cells leading to gene expression and liberate inflammatory mediators (IL-6 and IL-8) and upregulation of WBC-endothelial ICAM-1, vascular adhesion molecule-1 (VCAM-1), and E-selectin [74]. Likewise, MPs released following platelet activation may magnify WBC aggregation and assembly* via* the expression of P-selectin, adding further inflammation and buildup of atheroma plaque [31]. Similarly, platelet-derived MPs bind to neutrophils to cause neutrophil aggregation and enhance phagocytic activity, a common event seen after vascular inflammation [32]. Recently, it was revealed that monocyte-acquired MPs have exhibited the induction of oxidative stress marker (superoxide anion production), inflammatory cytokine release, and activation of NF-*κ*B gene in monocytes [44]. Taken together, these results demonstrate that, in atherosclerosis, increased levels of MPs may intensify inflammation and vascular injury.

### 6.6. EMPs Induced Cell Adhesion Molecules

It is well established that EMPs carry many surface molecules from the origin cell to show physiological function. A common such molecule is cell adhesion molecule which includes platelet endothelial cell adhesion molecule-1 (PECAM-1), VCAM-1, and ICAM-1 [47]. A crucial event of atheroma formation involves the adhesion of circulating monocytes on the vascular endothelium mediated by these cell adhesion molecules. A study showed that MPs and exosomes from platelets collectively enhance endothelial cell ICAM-1 and the adhesion of monocytic U937 cells to human umbilical vein endothelial cells [30]. Based on the type of stimulus for endothelial cell injury, either apoptosis or activation, the surface antigens molecules of EMPs are varied. Jimenez et al. investigated that increased amounts of the surface antigens E-selectin, ICAM-1, and VCAM-1 are present on EMPs obtained from induced endothelial cells, whereas in the case of EMPs derived from apoptotic endothelial cells less amounts of those surface antigens were present. Also, VE-cadherin, PECAM-1, and endoglin are at minimum levels on EMPs acquired from induced ECs [10, 39, 75, 76]. MPs derived from* in vitro* assay also induce cell adhesion molecules expression, specifically ICAM-1, at the surface of endothelial cells [30, 37, 77]. Likewise in* in vivo* human study, MPs isolated from human atherosclerotic plaques enhance endothelial cell expression of ICAM-1 following monocytes attachment to the intimal layer, but MPs derived from plasma possess no such function. The mechanism of enlarged endothelial ICAM-1 prompt by atherosclerotic plaque EMPs follows the conveyance of this cell adhesion molecule in a phosphatidylserine-dependent pathway from MPs to endothelial cells [78]. The role of atherosclerotic plaque MPs on the recruitment of inflammatory cells mediated by ICAM-1 might be distinctly applicable at the level of blood vessel ongoing advanced atherosclerotic lesions, since neointimal highly express VCAMs in comparison to luminal arterial endothelium [79]. These unusual neovessels are featured by random division of vessel branch having immature endothelial lining with altered permeability [80, 81]. For this reason, atherosclerotic plaques MPs bearing ICAM-1 may disperse along with blood flow and hence carry ICAM-1 to the surface of endothelial cell in “paracrine” manner [78].

## 7. Clinical Relevance of EMPs on Disease Related to Atherosclerosis

A number of diseases or physiological conditions like obesity and menopause associated with circulating MPs are also related to atherosclerosis progression. In menopausal women, the decrease level of endogenous estrogen possesses risk for vascular inflammation as the normal level of endogenous estrogen helps to maintain the anti-inflammatory, anticoagulant, and antithrombotic potency of endothelium. The mechanism behind this include balancing NO production, lowering oxidative stress, and decreasing the expression of procoagulant cell adhesion molecule [82]. High level of cellular MPs and proinflammatory cytokine in postmenopausal women leads to alterations of these cell adhesion molecules facilitating leukocytes and platelets adhesion to the endothelial surface thereby developing intimal thickening and vascular lesion [83]. So, it is likely that women at menopause are going through high risk of metabolic syndrome (MS) responsible for cardiovascular disease. Muthuvel Jayachandran et al. hypothesized that the changes in platelet function and levels of MPs obtained from blood and vascular cell during early menopause may reveal a link to the early atherothrombotic progression and found that, among recently menopausal women, specific platelet functions and level of cell membrane-derived procoagulant MPs alter individual components of MS. These alterations in cellular level clearly highlight how menopause contributes to MS and finally cardiovascular disease including atherosclerosis [84].

Obesity and type 2 diabetes patients are at increased risks of atherosclerosis and other cardiovascular complications since MPs are raised in blood plasma of diabetic, obese, and insulin-resistant individuals. An* in vivo* study done in 74 patients undergoing total knee arthroplasty reveals that the level of EMPs was higher in obese patient as compared to nonobese one. The level of EMPs was raised promptly after surgery, while 3 days after operation EMPs were restored to the basal level as before operation except in the case of obese patients. Furthermore, endothelial progenitor cells were less in obese patients, whereas the level of adipokines was raised after surgery. This suggests a vascular inflammatory condition that exacerbates adipokines level after the operation and may worsen endothelial repair [85]. An animal model to study the various pathways followed by MPs to promote atherosclerosis by augmenting endothelial dysfunction and upregulating release of proinflammatory mediators is high-fat diet (HFD) fed rats for prolonged period to make them obese and insulin resistant. Heinrich et al. [86] found that total MPs were drastically raised after feeding rats with HFD compared to normal basal diet. In this study, significantly high levels of MPs were obtained from endothelial, leucocyte, and platelet. MPs isolated from HFD fed rats possess the ability to stimulate ROS generation as well as expression of VCAM-1, an indication of a proinflammatory nature of MPs in this model.

## 8. Conclusions

Endothelial microparticle release is triggered by various stimulations followed by the activation of various pathways that can collectively make progress in the atherogenesis. Most importantly, oxidative stress, endogenous molecules (TNF-*α*, PDGF, and interleukin), and apoptosis inducer are responsible for EMPs release to undergo functional change in blood vessel eventually leading to the pathology of atherosclerosis. It is worth noting that various physiological and pathological conditions like menopause, obesity, and diabetic can promote atherosclerosis by generation of MPs. Furthermore, monocyte-macrophage-derived MPs are also critical part of the progression of unstable atherosclerotic plaques. Hence, medicine targeting the downregulation of EMPs as well as other MPs release can be a possible therapeutic option treating vascular inflammatory disease like atherosclerosis.

## Figures and Tables

**Table 1:** The types of microparticles and their role in disease progression.

Microparticles	Role in disease progression	Reference
PMP	Coagulation, inflammatory processes, thrombosis, and tumor progression	[19–21]

EMP	Endothelial dysfunction, angiogenesis, tumor growth, and increased oxidative stress	[11, 22–24]

MMP	Endothelial dysfunction, sepsis, and vascular inflammation	[25–27]

PMP: platelet microparticles; EMP: endothelial microparticles; MMP: monocyte microparticles.

**Table 2:** Scientific work on microparticle in association with its link with atherosclerosis.

Microparticles	Role in atherosclerosis	Reference
PMPs	Enhance cyclooxygenase-2 (COX-2) and intracellular adhesion molecules-1 (ICAM-1) release.	[28]
	COX-2 expression in monocytes through translocation of protein kinase C (PKC) from cytosol to the cell membrane.	[29]
	Enhance the adhesion of monocyte to human umbilical vein endothelial cells.	[30]
	Increase WBC aggregation and assembly *via *expression of P-selectin.	[31]
	Neutrophil aggregation and enhance phagocytic activity.	[32]

EMPs	CD-144/vascular endothelial cadherin positive.	[33]
	Reactive oxygen spices (ROS) formation.	[34]
	Matrix metalloproteinase-2 (MMP-2) activation and vascular matrix remodeling.	[35, 36]
	Reduce expression of p38-MAPK in human aortic endothelial cells caused reduction of TNF-*α* induced EMPs.	[37]
	EMPs exhibit the TF activity in atherosclerotic plaques.	[38]
	Expression of E-selectin, ICAM-1, and VCAM-1.	[39]

MMPs	Inducible nitric oxide synthase (iNOS) activation.	[40]
	Extracellular signal-regulated kinases (ERK1/2) activation.	[41]
	Procoagulant substances responsible for endothelial dysfunction.	[25]
	IL-1*β* in MMPs can enhance the vascular inflammatory.	[42]
	Atherosclerotic plaque formation in the ApoE^−/−^ mice.	[27]
	Increased expression of apoptosis regulator Bax, caspase-8, caspase-3, and cytochrome C in cardiomyocytes.	[43]
	Induce superoxide anion production, inflammatory cytokine release, and activation of NF-*κ*B gene in monocytes.	[44]
